# SCIRT lncRNA slows the formation of tumour initiating cells in breast cancer

**DOI:** 10.18632/oncoscience.537

**Published:** 2021-06-03

**Authors:** Alex de Giorgio, Leandro Castellano

**Affiliations:** ^1^ BenevolentAI, Bloomsbury, London W1T 5HD, United Kingdom; ^2^ University of Sussex, School of Life Sciences, Falmer, Brighton BN1 9QG, United Kingdom

**Keywords:** lncRNA, breast cancer, tumorigenesis

Breast cancer recurrence and chemoresistance are linked to populations of tumour initiating cells (TICs), which proliferate slowly and are often therapy resistant [1]. TICs exist in dynamic equilibrium with their differentiated progeny. How the transition to and from this cell state is regulated has been unclear, and a better understanding could lead to therapies that disrupt the transition or specifically target the TIC subset [2]. Zagorac et al. characterise a novel, conserved long noncoding RNA (lncRNA) regulating this transition named Stem-Cell Inhibitory RNA Transcript (SCIRT) [3, 4]. SCIRT’s function is unusual, counteracting the formation of the TIC state, despite being strongly induced when TICs form [4]. Its mechanism, explored further below, combines transcriptional control with antagonism of well-known protein regulators, seeming to act as a cellular brake to allow TIC formation to proceed at an appropriate pace [4].

Since 2005, when RNA-sequencing revealed the pervasive transcription of thousands of long noncoding RNAs (lncRNAs), many have been shown to be functional and to act by a wide variety of mechanisms [5]. One particular feature of lncRNAs is their relative tissue specificity relative to protein coding messenger RNA (mRNA) [6]. This has been linked to a capacity of lncRNAs to dynamically regulate the diversity of cell types and cell states found in complex organisms [6]. Zagorac et al. set out to explore the landscape of lncRNAs induced or suppressed during formation of breast tumourspheres at 16 hour and 5 day timepoints, a model of breast TIC formation [4].

LncRNAs have been shown to promote or restrain tumorigenesis and pluripotency in many cases [7, 8]. Yet inhibitory lncRNAs are usually downregulated during tumour progression, while oncogenic lncRNAs are upregulated as they enhance oncogenic processes. That SCIRT is upregulated in more aggressive cancers, and the TIC subset, is unusual, and suggests that the orchestration of transcriptional events during the cell state transition may leave cells unviable if it proceeds too quickly.

Zagorac et al. identify SCIRT through its marked upregulation at both 16h and 5d timepoints [4]. Not only is SCIRT evolutionarily conserved, an important marker of lncRNA functionality, but silencing SCIRT reduced in a dramatic increase in mammosphere formation, and tumour initiation in an in vivo xenograft model [4].

The mechanism by which SCIRT acts involves certain well known transcriptional regulators. EZH2 has been shown to be highly expressed in tumour initiating cells of many cancer types, playing roles in their expansion and maintenance [9], while SOX2, a central regulator of embryonic stem cell pluripotency, has been shown to increase stemness while decreasing cell cycle in neural stem cells [10]. Zagorac et al. show that EZH2 not only increases stemness-related transcription, but also reduces cell cycle transcription. These prior examples suggest that these two factors could also act in breast TICs cells to slow cell proliferation. This is in line with the role of SOX2 in neuronal progenitors and the fact that SOX2 and EZH2 cooperate in embryonic stem cells [11].

During mammosphere formation, SCIRT is not only highly induced, but remains closely chromatin associated, and binds to both EZH2 and FOXM1, antagonising their abilities to transcribe a multiple regulators of stemness and other processes. The authors find SCIRT to bind to over enhancers or promoters of over 7000 genes. The observation that SCIRT binds both EZH2, and FOXM1, suggests a complex interplay with these regulators may take place [4]. SCIRT appears to oppose EZH2 and SOX2’s transcriptional programs by recruiting the cell cycle master transcription factor FOXM1 to cell cycle promoters. Recruiting FOXM1 increases transcription of cell cycle genes that were shown to be repressed by EZH2 and SOX2 and counteracts the activity of EZH2 and SOX2 on stemness promoters. High overlaps between sites bound by SCIRT, and those bound by these proteins were observed, combined with opposite effects from these bindings, since SCIRT silencing upregulated genes that were reduced by EZH2 silencing and vice versa [4]. 

Recently, reordering of RNA-sequenced single cells in ‘pseudotime’ has revealed the delicate sequence of transcriptional bursts that takes place during cell differentiation [12]. In this context, Zagorac et al.’s identification of a cellular brake, seeming to act to avoid the over-hasty progression of a cell state transition is an intriguing finding. It suggests that future examples may emerge by looking for molecules whose expression correlates with but whose function antagonises that of important regulators. Given the interest in the field in disrupting TIC function, SCIRT and other such cellular brakes may represent interesting targets to compromise the ability of tumours to safely transition between cell states to sustain their growth and progression.
